# The Physician’s Pocket Dose and Symptom Book

**Published:** 1857-07

**Authors:** 


ART. VI.—*The Physician's Pocket Dose and Symptom Book;* Containing the Doses and Uses of all the Principal Articles of the Materia Medica and Chief Officinal Preparations, &c. &c. By JOSEPH H. WYTHES, A.M., M.D. Second Edition. Lindsay & Blakiston, 18mo., pp. 230, Philadelphia, 1857.

WE belong to that class of men who have, as a general thing, no respect for the small writings of the profession; and yet we recollect, with what pleasure we carried about with us, in our pocket, when we were a student, a copy of Fyfe's Anatomy, and with what avidity we read, as we were waiting for the tardy steps of our master, the author's account of the brain, heart, and other organs, treasuring up items in our mind as food for subsequent digestion as we again emerged into the open street. Even our Sabbath meditations were sometimes seriously interrupted by a dip of this kind. Who is there among us, who has not occasionally longed for the *vade-mecum*—the little go-with-me—that he might refresh his memory upon some practical subject, as he was riding through the crowded thoroughfares of a large city, or proceeding on horseback over the tedious and muddy roads of the country, with no flowers to greet his sight, or birds to charm his ear with their sweet warblings? It is at such times that the medical student—we do not mean the under-graduate, but the young and aspiring practitioner—finds genuine comfort and consolation in this class of productions, and feels inclined to hug them with a sort of parental tenderness to his bosom. He is suddenly summoned to a case of poisoning with arsenic or opium, to one of flooding, or to one of compound fracture. He has no time to refer to the more elaborate works in his library; the case is too urgent to admit of delay; and yet he feels that he ought to refresh his memory respecting the diagnosis and treatment of the disease to which he is so peremptorily called. Here is a dilemma. He cannot put the ponderous octavo into his pocket, or hold it in his hand as he drives or rides along at a furious rate to see his patient. No! But he snatches up his faithful, never-failing *vade-mecum*, his *multiparval* friend, and, in a moment, his eye rests upon the very spot that he wishes to find. He takes a little draught; his thirst is allayed; and he proceeds on his way, thankful that he has not despised the day of little things.

Why, reader, the very word, *vade-mecum*, has something cosy and pleasant about it; go with me, be my companion, my friend; a friend in need, a friend indeed! Such books, if properly executed, are like keys to the locks of the great storehouse of knowledge—*cul-de-sacs*, filled with the essence and fragrance of the great books of the profession.

Dr. Wythes, who is favorably known as the author of several small works of merit, has performed his task well. He possesses the faculty of condensation in a remarkable degree, and his little work does not contain one ambiguous expression. The book is, as it professes to be, nothing but a pocket remembrancer; but, as such, we regard it as one of great value, a circumstance fully attested by the early demand for a second edition. It comprises a table of weights and measures, rules for proportioning doses, a list of common abbreviations, a table of poisons and antidotes, a classification of the materia medica, an alphabetical list of the principal medicinal articles and preparations, a table of symptoms of disease, and a brief outline of general pathology and therapeutics. Of all the little works of the kind now extant, we know of none that is equal to it.

